# Sensorimotor inhibition during emotional processing

**DOI:** 10.1038/s41598-022-10981-8

**Published:** 2022-04-29

**Authors:** Alessandro Botta, Giovanna Lagravinese, Marco Bove, Elisa Pelosin, Gaia Bonassi, Alessio Avenanti, Laura Avanzino

**Affiliations:** 1 Department of Experimental Medicine (DIMES), Section of Human Physiology, University of Genoa, Viale Benedetto XV/3, 16132 Genoa, Italy; 2 Department of Neuroscience, Rehabilitation, Ophthalmology, Genetics and Maternal Child Health (DINOGMI), University of Genoa, Genoa, Italy; 3 IRCCS Ospedale Policlinico San Martino, Genoa, Italy; 4 S.C. Medicina Fisica e Riabilitazione Ospedaliera, ASL4, Azienda Sanitaria Locale Chiavarese, Chiavari, Italy; 5 Centro di Neuroscienze Cognitive and Dipartimento di Psicologia, Campus Cesena, Alma Mater Studiorum-University of Bologna, Cesena, Italy; 6 Centro de Investigación en Neuropsicología y Neurociencias Cognitivas, Universidad Católica del Maule, Talca, Chile

**Keywords:** Neuroscience, Emotion, Sensorimotor processing

## Abstract

Visual processing of emotional stimuli has been shown to engage complex cortical and subcortical networks, but it is still unclear how it affects sensorimotor integration processes. To fill this gap, here, we used a TMS protocol named short-latency afferent inhibition (SAI), capturing sensorimotor interactions, while healthy participants were observing emotional body language (EBL) and International Affective Picture System (IAPS) stimuli. Participants were presented with emotional (fear- and happiness-related) or non-emotional (neutral) EBL and IAPS stimuli while SAI was tested at 120 ms and 300 ms after pictures presentation. At the earlier time point (120 ms), we found that fear-related EBL and IAPS stimuli selectively enhanced SAI as indexed by the greater inhibitory effect of somatosensory afferents on motor excitability. Larger early SAI enhancement was associated with lower scores at the Behavioural Inhibition Scale (BIS). At the later time point (300 ms), we found a generalized SAI decrease for all kind of stimuli (fear, happiness or neutral). Because the SAI index reflects integrative activity of cholinergic sensorimotor circuits, our findings suggest greater sensitivity of such circuits during early (120 ms) processing of threat-related information. Moreover, the correlation with BIS score may suggest increased attention and sensory vigilance in participants with greater anxiety-related dispositions. In conclusion, the results of this study show that sensorimotor inhibition is rapidly enhanced while processing threatening stimuli and that SAI protocol might be a valuable option in evaluating emotional-motor interactions in physiological and pathological conditions.

## Introduction

Appropriate motor responses to potential threats require rapid evaluation of the menace’s features and the surrounding environment^1^. Being able to direct attention toward relevant sensory stimuli, as well as to correctly read emotional signals in others, is crucial in order to plan and consequently choose the most appropriate reaction^2–5^. Threat-related visual stimuli are rapidly processed by the brain as shown by early modulations of event-related potentials (ERPs)^4,6,7^; these stimuli are thought to increase sensory vigilance and grab attention^2,8,9^. In turn, perceived threats rapidly prime the body for action, as shown by both behavioural and electrophysiological evidence^10,11^. Rapid motor responses to emotional stimuli have been often reported using motor-evoked potentials (MEPs) induced by transcranial magnetic stimulation (TMS) of the primary motor cortex (M1), which offers the possibility to probe the motor system with high temporal resolution^12–14^.

These studies have shown that emotional body language (EBL)—and in particular, threat-related expressions, such as fearful body postures—modulate corticospinal excitability (CSE) and intracortical M1 processes such as intracortical facilitation (ICF)^12–15^. Also, emotional stimuli like emotional facial expressions or more complex stimuli like natural emotional scenes from the International Affective Pictures System (IAPS) have been shown to modulate CSE^16–22^. These studies have often reported two types of motor modulations, i.e., early inhibitory effects (i.e. when CSE or ICF was probed 70–150 ms after stimulus onset) that are thought to reflect early freezing-like orienting to the possible source of threat and later (> 150 ms) motor facilitations, reflecting increased motor readiness to emotional stimuli^12,15,18,20,23,24^.

Motor preparation requires fine tuning of sensorimotor interactions between somatosensory and motor regions^25,26^. However, despite the relevance of sensorimotor integration to efficient action processing^26–28^, to date, little is known about how the underlying sensory-to-motor interactions are affected by emotional information in humans.

Sensorimotor system has been shown to be sensitive to emotions to the extent that emotional recognition processes are able to induce phenomena of motor mimicry and somatosensory simulation, which activate the motor and the sensory system^29,30^. Furthermore, it has also been shown that these effects are more evident when the observer is confronted with negative emotions (e.g., fear or anger) which are able to trigger similar internal emotional states and in turn enhance sensorimotor system’s readiness^30^.

A valuable method to non-invasively probe sensorimotor integration at the cortical level is the so called short-latency afferent inhibition (SAI) TMS protocol^31^ which involves pairing electrical stimulation of peripheral somatosensory afferents with focal TMS targeting of the contralateral M1.

Studies using the SAI protocol have shown that peripheral nerve stimulation at the contralateral wrist reduces the amplitude of MEPs when the TMS pulse is given to M1 2–8 ms after the arrival of the afferent volley in cortex. The circuitry involved in SAI is complex. The sensory input exerts its inhibitory effects on the corticospinal neurons through γ-Aminobutyric acid (GABA)-ergic intracortical circuits^32,33^, but its magnitude is also modulated by dopaminergic^34^ and cholinergic neuromodulatory circuits^35^.

As previously stated, although the exact mechanisms of SAI are still not clear, the contributions of several neurotransmitters in the phenomenon have been studied. It has been shown by Di Lazzaro et al. that SAI was reduced (i.e., less sensorimotor inhibition) when subjects received benzodiazepines binding the α1-subunit of the GABA_A_ receptors (e.g., Zolpidem)^36^, while it was found to be increased after the administration of acetylcholinesterase inhibitors^37^. Thus, as suggested in a review by Turco et al., SAI generation is probably related to specific inhibitory cells activated by cholinergic inputs^38^. It has been also shown that SAI was reduced in PD patients under l-Dopa medication in the ON phase, showing a potential detrimental effect on sensorimotor inhibition of high concentration of Dopamine^34^. If we consider the fact that cholinergic efferent projections of the basal forebrain nuclei to the cortex play a critical role in functions such as arousal, attention, cognitive and emotional processes^39^ and that SAI has been shown to be dynamically modulated by cognitive processes, for instance working memory and attention^40–42^ and planning of both executed and imagined movements^43,44^, it appears plausible that it is a suitable tool to explore dynamic modulation of cholinergic networks during higher-order functions such as emotional processing.

Here, we designed two TMS experiments to assess SAI while healthy participants were observing fear-related and happiness-related EBL and IAPS pictures and neutral controls. Our hypothesis is that processing emotional signals—which are known to affect perceptual and motor as well as cognitive processes—would also influence sensorimotor integration. While prior work exploring early motor response to emotional stimuli have typically used EBL stimuli—which are known to induce emotion-related but also motor resonance effects at the CSE level^12,19,45,46^—in the present research we tested both EBL and IAPS pictures so to assess the generalizability of the effects and disentangle whether any modulation of the SAI protocol could be related to emotional modulation, motor resonance or other general effects. More specifically, we aimed to compare complex emotional scenes, controlled in order to be embedded with minimal motor information, with emotional body postures, whom intrinsically carry both motor and emotional information, in order to understand if the prevalence of one of these two types of information (i.e., emotional or motor) is responsible for the modulation of the sensorimotor network.

Building on prior work showing early and later changes in MEPs during emotional processing (e.g., Refs.^12,13,23,47^), here, we tested SAI at two different time-points, i.e., at 120 ms and 300 ms from picture onset (as performed by Borgomaneri et al. in the studies^12,23^, respectively). While the 120 ms time point allows us to assess early subcortical-cortical automatic response to emotional stimuli^12^, the 300 ms timepoint allows assessing later, cognitive processing of motor and emotional information^12,23^. Building on the evidence that threat-related signals triggers early response in cortico-subcortical networks involved in emotion processing and action planning^48–50^ and that early response to emotional signals—in particular to fear-related signals—may involve inhibitory motor processes^12,13,23^, we expected that fear-related pictures would enhance SAI effects in the early time point.

A control experiment was also performed to test whether a simple, non-emotional, visual stimulus (i.e., while observing a black screen at rest) was able to modulate SAI at 120 and 300 ms differently from the SAI recorded at baseline. In this case, we aimed to exclude that the modulation of sensorimotor inhibition could have been a consequence of the mere observation of a visual stimulus itself, possibly driven by light changes and not by the increased attentional load derived from the observation of more complex emotional stimuli.

Finally, we also considered the fact that motor reactivity during perception of social and emotional information can be largely affected by inter-individual differences in specific personality traits^22,51,52^. In particular prior work has shown an association between behavioural inhibition (BIS) and the magnitude of motor response to EBL, with larger M1 suppression due to fear-related EBL in participants showing higher scores at the BIS scale^15^. Therefore, we also submitted participants to BIS and behavioural approach system (BAS)^53,54^. The BIS is described as an attentional system that disrupts ongoing behavioural response in order to allow the subject to focus on relevant cues (e.g. punishment, non-reward or novelty), while the BAS is linked to positive feelings such as joy or optimism or negative such as rage and aggression; and BIS/BAS scales have been used in several neuroscientific studies^55,56^. In this way, we tested whether interindividual differences in BIS/BAS predict magnitude of sensorimotor integration as indexed by the SAI effects.

## Methods

### Participants

Thirty healthy participants (15 males, mean age ± SD 23.7 years ± 3.3) were enrolled in the main study (16 for the EBL experiment and 14 for the IAPS experiment) and 10 additional participants (6 males, 28.3 years ± 2.1) took part in a control experiment in which sensorimotor modulation was investigated during observation of a non-emotional visual stimulus (see “Visual stimuli”).

In the main study, 16 participants (6 males, mean age ± SD 24.6 years ± 3.9) were tested with EBL stimuli and 14 participants (9 males, mean age ± SD 22.5 years ± 2.3) were tested with IAPS stimuli. All participants were in good health, without any nervous, muscular, orthopaedic or cognitive disorders. Right arm dominance was determined by means of the Edinburgh Handedness inventory^57^. Prior to the experimental session, all participants gave written informed consent and filled out a TMS safety, 13-items screening questionnaire^58^. The experimental protocol was approved by the ethics committee of the University of Genoa and was carried out in agreement with legal requirements and international norms stated in the adjourned declaration of Helsinki^59^.

Considering the impossibility to retrieve previous experimental SAI data recorded in a similar study design, in the main experiment we opted for a post-hoc power analysis in order to assess if the sample size we used in our study was appropriate. Results of the post-hoc power analysis showed that the sample size used for the EBL experiment (N = 16) was appropriate with a power of 95% (1 − β = 0.95; α = 0.05; effect size = 0.97), while for the IAPS experiment (N = 14) we retrieved a power of 94% (1 − β = 0.94; α = 0.05; effect size = 1.00). Power analysis was computed on G*Power 3.1.

### Visual stimuli

Visual stimuli were presented on a 22-inches computer screen (resolution 1680 × 1050, refresh rate 60.0 Hz; 16.67 ms ± 12.37 ms) located at 1.5 m away from the participants. Refresh rate was assessed via a photosensor and corresponded to normative values^60^.

**Figure 1:**
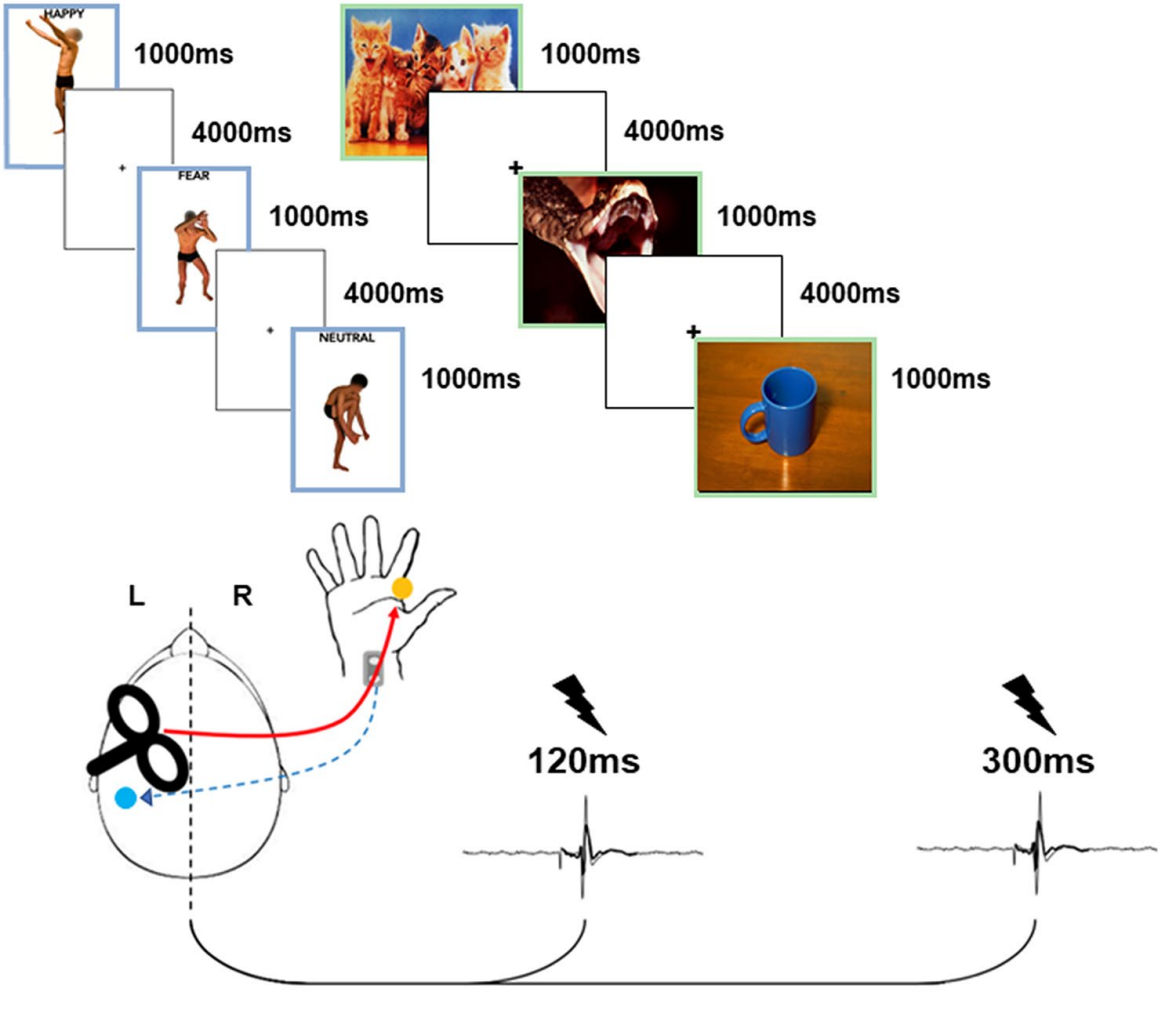
Experimental design. SAI was tested via an electrical stimulus (ES) delivered over of the median nerve (dashed blue arrow), followed 20 ms later by a transcranial magnetic stimulus (TMS) stimulus (red arrow) over the left first dorsal interosseus (FDI) cortical area at two timepoints, 120 and 300 ms after visual stimulus onset (black lightning). The blue dot indicates the somatosensory afferences to the sensorimotor cortex, while the yellow dot represents the FDI muscle. Each visual emotional stimulus had duration of 1000 ms, interspersed by a blank, white fixation screen of 4000 ms, for a total of 45 stimuli for each trial [emotional body language (EBL) and International Affective Picture System (IAPS)]. The same experimental design was followed also for the control experiment, with the emotional visual stimuli replaced by a black cross on a white screen.

A total of 90 emotional visual stimuli were used for the main study, 45 for the EBL experiment and 45 for the IAPS experiment (see Supplementary Materials and Fig. 1). For the EBL experiment, emotional posture pictures were selected from a validated EBL database^12,19^. EBL pictures depict four actors in different postures with emotional and neutral valence, thirty depicting negative (fear) and positive (happiness) movements and fifteen with no emotional significance (neutral). The actors were not handling objects and their face was blanked-out.

Regarding the IAPS experiment, 45 stimuli were taken from the IAPS database, fifteen with negative emotional valence (fear), fifteen with positive valence (happiness) and fifteen neutral pictures (neutral). Fear evoking IAPS pictures were extrapolated from the study of Barke and colleagues in order to select fear-related stimuli, not contaminated by other negative emotions (e.g. disgust or rage)^61^.

Furthermore, considering that the goal of the IAPS experiment was to test whether emotional scenes, with little or no motor information, were able to induce a modulation in the sensorimotor circuit, we excluded all the IAPS pictures that depicted whole human bodies involved in some kind of actions.

For the control experiment, the visual stimulus was a black cross on a white screen, with controlled resolution and refresh rate as for the main experiment.

Each experimental stimulus (EBL and IAPS pictures in the main experiment, black cross in the control experiment) lasted 1000 ms, with an inter-stimulus interval of 4000 ms.

### Transcranial magnetic stimulation (TMS)

Single-pulse TMS was delivered using a Magstim 200 stimulator (Magstim, UK) with a monophasic current waveform connected to a figure-of-eight-shaped coil (external diameter of each loop, 9 cm) held tangentially to the scalp. The centre of the junction of the coil was placed over the hand area of the contralateral M1 (i.e., left M1) at the optimal position (hotspot) to elicit MEPs in the right first dorsal interosseous (FDI), with the handle pointing backwards and a 45° away from the midline. With this coil orientation, the induced current flowed in an anterior–medial direction approximately perpendicular to the central sulcus. The optimal coil location was searched by slightly moving the coil over the left M1 area until MEPs of maximal amplitude and lowest threshold in the right FDI were elicited. The exact coil position was marked by an inking pen to ensure an accurate positioning of the coil throughout the experiment.

### Short latency afferent inhibition (SAI) protocol

SAI was tested following the validated protocol by Tokimura and colleagues^31^.

The SAI effect was tested with a supra-threshold test TMS stimulus over the M1 representation of right FDI adjusted to produce MEPs of ~ 1 mV (peak-to-peak amplitude), preceded (20 ms inter-stimulus interval) by an electrical conditioning stimulus over the contralateral median nerve at the right wrist. This conditioning interval was shown to be optimal to evoke MEP inhibition in several studies^31,62^. Electrical stimulation (ES) was delivered through a bipolar electrode (cathode proximal) using a square-wave pulse (duration, 200 μs) set at intensity just above the threshold for evoking a small twitch in the *opponens pollicis* muscle (Digitimer D180 high voltage electric stimulator). In each experiment (EBL, IAPS) of the main study, a total of 90 trials were performed, of which 45 were conditioned trials (COND: ES + TMS) and 45 unconditioned trials (TEST: TMS). In the control experiment, a total of 30 trials were performed (15 TEST and 15 COND). For all conditions, in the main experiment and in the control experiment, SAI was tested at two time-intervals: 120 ms and 300 ms after visual stimulus onset. Additionally, two baselines recording of 30 trials each were recorded before (PRE) and after (POST) the experimental session.

### Electromyographic (EMG) recording

EMG was recorded with silver disc surface electrodes placed with a belly-tendon montage over the right FDI muscle. Electromyography signals were amplified and filtered (20 Hz to 1 kHz) with a D360 amplifier (Digitimer). The signals were sampled at 5000 Hz, digitized with a laboratory interface (Power 1401, Cambridge Electronic Design, Cambridge, UK) and stored on a personal computer for display and later offline data analysis.

### Experimental procedures

In all experiments, participants were comfortably sitting on a chair at a distance of approximately 1.5 m from the computer screen where the visual emotional or neutral stimuli (main experiment) or the non-emotional stimulus (control experiment) were presented. The experiment was programmed using Matlab (version 9.9.0.1467703, MathWorks, Natick, MA, USA) software to control picture presentation and to trigger ES and TMS.

MEPs were collected in 4 blocks (two baseline blocks and two experimental blocks). The baseline blocks were the first (PRE) and the last (POST) (30 trials each): participants were asked to keep their eyes open with the instruction to watch a black screen while receiving TMS over M1 (interpulse interval 4000 ms). The interpulse interval was determined following the Guidelines of the International Federation of Clinical Neurophysiology^63^. Fifteen COND trials and 15 TEST trials were collected in each block.

In the experimental blocks (90 trials each) participants were asked to stay still and focus on the pictures sliding on the screen. In these two blocks, TMS pulse was delivered at 120 or 300 ms after picture onset and the order of the blocks was randomized. The trials in the experimental blocks were divided in 45 COND trials and 45 TEST trials, which in turn provided for 30 emotional stimuli (15 for fear and 15 for happiness) and 15 neutral stimuli each.

Participants were asked to stop the experiments whenever they felt discomfort or pain. They were also invited to maintain attention to the stimuli at regular intervals throughout the experiment and a verbal comment was asked at the end of each trial were all participants had to tell if they perceived any emotion on the observed pictures. A pause of 3–4 min between blocks was allowed to prevent fatigue or lack of attention. The experiment lasted for approximately 120 min.

A similar protocol was used for the control experiment, where 30 trials (15 COND and 15 TEST) were performed for the two baseline blocks (PRE and POST) and 30 trials were performed at 120 and 300 ms after stimulus onset.

Finally, the Italian version of the BIS and BAS questionnaire was submitted via email to all main experiment’s participants in order to acquire individuals’ specific personality dimensions^54^. The whole questionnaire consists of 20 items of which the firsts seven give the BIS score (maximum 35 points) and the remaining give the BAS score (maximum 65 points). The BAS scale is divided in three main subclasses as follow: BAS Reward from item 8 to item 12, BAS Drive from item 13 to item 16 and BAS Fun from item 17 to item 20. The subject had to give a rating from 1 to 5, where 1 stood for “It doesn’t describe me at all” and 5 stood for “It describes me completely”. A high score in the BIS scale indicates an increased propensity in withdrawal or inhibitory behaviour when facing a potential threat, while a high score in the BAS scale is an indication of action engagement^55,64^.

### Data analysis

Neurophysiological data were analysed offline. The mean peak-to-peak MEP amplitude (in mV) was computed for each condition, with or without peripheral stimulation (MEPs COND and MEPs TEST). To evaluate the SAI effect, conditioned MEPs were expressed relative to unconditioned MEPs (SAI = COND/TEST). Moreover, SAI value at each testing time (120 and 300 ms) was normalized to SAI recorded at baseline (SAI Ratio = SAI_Experiment_/SAI_Baseline_). This normalization shows data as an increased or decreased SAI compared to the SAI recorded at baseline. A value greater than 1 indicates a SAI reduction in relation to baseline (i.e., reduced sensorimotor inhibition), whereas a value lower than 1 indicates an increase of SAI in relation to baseline (i.e., increased sensorimotor inhibition)^44,65^. The same analysis was run also for the data acquired in the control experiment.

### Statistical analysis

Before performing further statistical analysis, we checked that all variables were normally distributed (Shapiro–Wilk test) and that sphericity was respected (Mauchly’s test). Levene’s test of homogeneity of variances was also performed in order to assess the comparability of the two datasets in the main experiment (EBL and IAPS).

The effects of electrical conditioning on MEP amplitudes in the two baseline blocks were tested using a mixed factors ANOVA with BLOCK (PRE and POST) and TYPE OF STIMULATION (TEST and COND) as within-subjects factors and GROUP (EBL and IAPS) as between-subjects factor.

For the main study, SAI Ratio data were analysed via a 3 × 2 × 2 mixed factors ANOVA, with EMOTION (fear, happiness and neutral) and TIME (120 ms and 300 ms) as within-subject factors and GROUP (EBL and IAPS) as between-subject effect. Post-hoc analysis was performed by means of Bonferroni correction method for multiple comparison.

We also performed a 3 × 2 × 2 mixed factors ANOVA, with the same within-subjects factors (EMOTION and TIME) and GENDER (Male and Female) as between-subjects effect, for both EBL and IAPS stimuli.

In order to assess whether there was a difference between the unconditioned MEPs amplitudes (MEPs TEST) and SAI effects (COND/TEST) recorded while processing EBL and IAPS pictures and the ones recorded at baseline, we performed a 3 × 2 mixed factors ANOVAs with CONDITION (Baseline, 120 ms and 300 ms) as within-subjects factor and GROUP (EBLs and IAPS) as between-subjects factor separately for each emotion (fear, happiness and neutral). We opted to name differently the TIME and CONDITION factors in order to highlight the presence of baseline data in CONDITION. Bonferroni corrected post-hoc tests were used to test significant effects.

In the control experiment, MEPs TEST and SAI data were analysed via a repeated measure ANOVA with CONDITION (Baseline, 120 ms and 300 ms) as within-subjects factor.

Finally, to investigate a possible relationship between SAI modulation and personality trait in relation to emotional processing, we performed a Pearson correlation between BIS/BAS questionnaire scores and SAI Ratio for normal distributed data and otherwise a Spearman’s Rho correlation test was run.

Statistical analysis was performed with SPSS 25. p-values of 0.05 were considered as threshold for statistical significance.

## Results

At baseline, when TMS was preceded by ES, MEPs amplitude consistently decreased relatively to TMS alone (Table 1), thus replicating the typical SAI effects. Accordingly, the statistical analysis showed a strong effect of TYPE OF STIMULATION (F [1, 28 = 324.567; p < 0.01; pη^2^ = 0.921), with lower amplitude in the COND condition (mean ± SEM = 0.468 ± 0.027) compared to the TEST condition (1.27 ± 0.048), while no influence of the factors BLOCK or GROUP were found (all F ≤ 2.23; all p > 0.05), thus suggesting no change in motor excitability over time in the two experimental groups submitted to the observation of EBL and IAPS stimuli. Furthermore, considering that no influence of BLOCK was observed, indicating no changes in motor excitability over time, the baseline blocks were averaged for computing the SAI Ratio (see next section).

**Table 1:** MEPs and SAI data in the main experiment. In the table, MEPs values for the Test and Conditioning stimuli and SAI data are reported for EBL (A) and IAPS (B). All values are reported as mean ± standard error of the mean (SEM).

A	EBL						
	Baseline	Fear		Happy		Neutral	
		120 ms	300 ms	120 ms	300 ms	120 ms	300 ms
MEP test stimulus (mV)	1.17 ± 0.23	1.07 ± 0.32	1.24 ± 0.41	1.03 ± 0.32	1.17 ± 0.38	1.03 ± 0.22	1.20 ± 0.38
MEP conditioning stimulus (mV)	0.44 ± 0.03	0.33 ± 0.05	0.50 ± 0.05	0.40 ± 0.06	0.50 ± 0.05	0.42 ± 0.06	0.52 ± 0.05
SAI	0.37 ± 0.03	0.31 ± 0.03	0.41 ± 0.04	0.39 ± 0.04	0.43 ± 0.03	0.40 ± 0.04	0.44 ± 0.04

B	IAPS						
	Baseline	Fear		Happy		Neutral	
		120 ms	300 ms	120 ms	300 ms	120 ms	300 ms
MEP test stimulus (mV)	1.35 ± 0.08	1.34 ± 0.14	1.31 ± 0.11	1.30 ± 0.12	1.36 ± 0.09	1.31 ± 0.12	1.34 ± 0.11
MEP conditioning stimulus (mV)	0.50 ± 0.05	0.37 ± 0.03	0.56 ± 0.07	0.50 ± 0.06	0.64 ± 0.09	0.51 ± 0.06	0.5 ± 0.07
SAI	0.38 ± 0.03	0.29 ± 0.02	0.44 ± 0.04	0.41 ± 0.04	0.46 ± 0.04	0.41 ± 0.04	0.46 ± 0.04

### Main experiment

#### Modulation of sensorimotor integration during perception of emotional pictures: comparison between emotions

**Figure 2:**
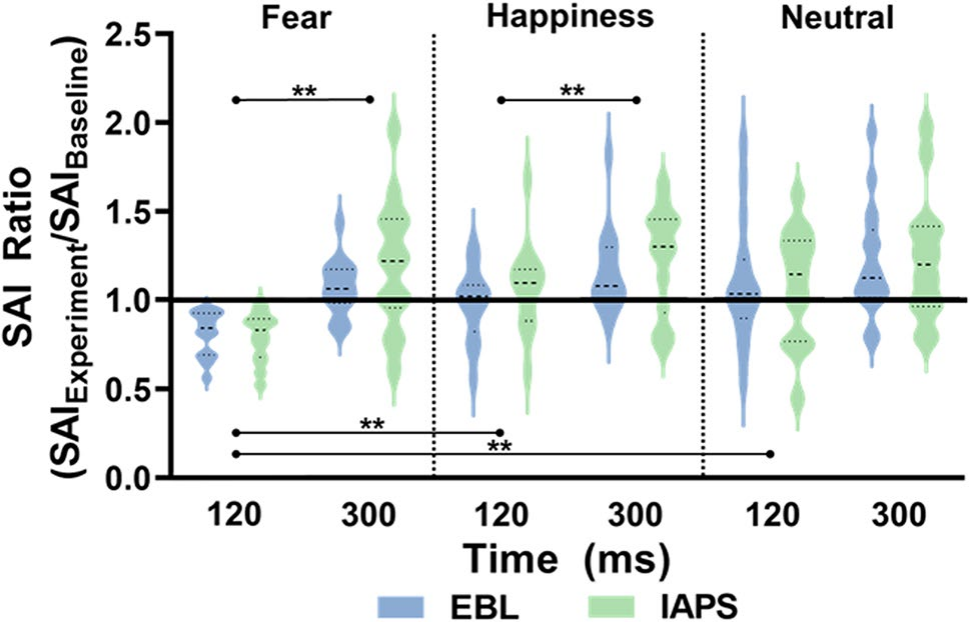
SAI Ratio data. The picture shows the violin plots of all SAI Ratio data for EBL and IAPS. A value greater than 1 (black thick line) indicates a decreased inhibition (SAI reduction) in relation to baseline, whereas a value lower than 1 indicates an augmented inhibition (SAI increase) in relation to SAI recorded at baseline. As observable, the only increase of SAI was at 120 ms after stimulus onset for fearful emotional stimuli. All SAI Ratio data computed at 300 ms showed a decreased SAI across emotional and neutral stimuli in both EBL and IAPS. SAI Ratio data are reported on the y-axis, while the two timepoints 120 and 300 ms are reported on the x-axis, separately for every condition. **p < 0.01.

Statistical analysis on SAI ratio values showed that sensorimotor modulation occurred at 120 ms specifically for fearful stimuli and at 300 ms for all the emotional stimuli (see Fig. 2). ANOVA on SAI Ratio data showed significant results for both main effects EMOTION (F [2, 56] = 9.723; p < 0.01; pη^2^ = 0.258) and TIME (F [1, 28] = 17.709; p < 0.01; pη^2^ = 0.387), which were qualified by the interaction effect EMOTION × TIME (F [2, 56] = 4.079; p = 0.035; pη^2^ = 0.127). All the effects involving the factor GROUP were not significant in the ANOVA (all F ≤ 0.508; all p > 0.05), suggesting comparable sensorimotor modulations with the two sets of pictures. Post-hoc analysis of the EMOTION × TIME interaction showed that only during processing of fearful stimuli at 120 ms from the stimulus onset SAI increased (i.e., larger inhibition) respect to the same timing for happiness (120 ms, p < 0.01) and neutral (120 ms, p < 0.01). Furthermore, only for fear and happiness, SAI recorded at 120 ms after stimulus onset was significantly higher compared with SAI recorded at 300 ms (p < 0.01 for fear; p = 0.012 for happiness).

No effects for GENDER was retrieved in the analysis for EBL or IAPS stimuli (all F ≤ 0.508; all p > 0.05).

#### Modulation of corticospinal excitability and sensorimotor integration during perception of emotional pictures

Considering that the analysis on SAI Ratio data showed an influence of EMOTION, which was different for the early and later time points considered (i.e., EMOTION × TIME interaction) we further investigated SAI modulation during observation of emotional stimuli performing additional analyses. The ANOVAs on MEPs TEST showed no significant main effects or interaction of the factors CONDITION or GROUP across the three emotions (fear, happiness and neutral; all F ≤ 1; all p > 0.05).

**Figure 3:**
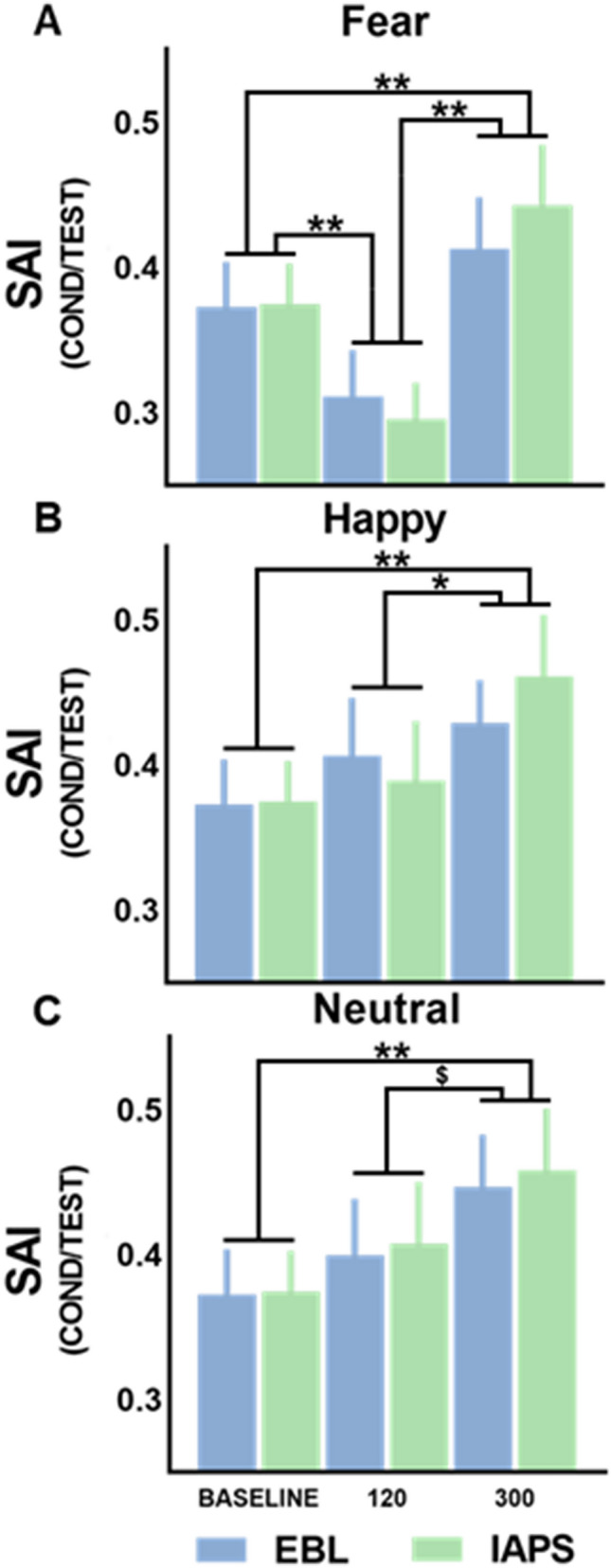
SAI data. All SAI data are graphically reported. In (**A**), the increase of SAI at 120 ms after stimulus onset for fearful stimuli is observable in both conditions (EBL and IAPS) in the comparison with SAI recorded at baseline (no visual stimuli). At 300 ms after stimulus onset, SAI was decreased compared to baseline for all emotional (**A**,**B**) and neutral stimuli (**C**) in both conditions (EBL and IAPS). SAI data (MEP conditioned/MEP test) are reported on the y-axis, while the three timepoints at which SAI was tested (Baseline, 120 ms and 300 ms) are reported on the x-axis. *p < 0.05, **p < 0.01, ^$^p > 0.05 (trend).

In contrast, the ANOVA on SAI data for fear showed significant effect of CONDITION (F [2, 56] = 28.814; p < 0.01; pη^2^ = 0.507), but no main effect or interaction involving the factor GROUP (p > 0.05). Post-hoc analysis (see Fig. 3A) showed that the SAI effect increased (i.e., lower SAI values) for fearful stimuli in the early time point (120 ms) relative to the later time point (300 ms) and baseline (all p < 0.01). Furthermore, at 300 ms after fearful stimuli onset, SAI was significantly lower relative to baseline (p < 0.01).

The ANOVA on SAI effects for the other two conditions (Happiness, Fig. 3B; Neutral stimuli, Fig. 3C) showed a significant effect of CONDITION for both happiness (F [2, 56] = 9.669; p < 0.01; pη^2^ = 0.257) and neutral stimuli (F [2, 56] = 5.824; p < 0.01; pη^2^ = 0.172), but not main effect or interaction involving the factor GROUP (all F ≤ 1; all p > 0.05). Post-hoc analysis showed that for both happiness-related and neutral stimuli, SAI significantly decreased at 300 ms relative baseline levels (all p < 0.01) and showed similar trend relative to the 120 ms condition (happiness-related stimuli: p = 0.016; neutral stimuli p = 0.082).

### Correlation analysis

Twenty-one out of 30 participants accepted to compile the BIS/BAS questionnaires and were included in the correlation analysis. Three of them were excluded as outliers (distance from the trend-line higher than 2.5 standard deviations), for a total of 18 participants from both the EBL and the IAPS experiments.

Since, statistical analysis on SAI Ratio data did not show any significant difference between groups (EBL and IAPS), before running the correlation analysis, we performed a linear regression analysis to verify that the GROUP was not a significant predictor for BIS, BAS and SAI RATIO data. Statistical analysis showed that GROUP was never a significant predictor (Supplementary Information), thus we run the correlation analysis combining the data of EBL and IAPS conditions.

Pearson correlation was run separately for the BIS and BAS questionnaire scores that presented normal distribution.

**Figure 4:**
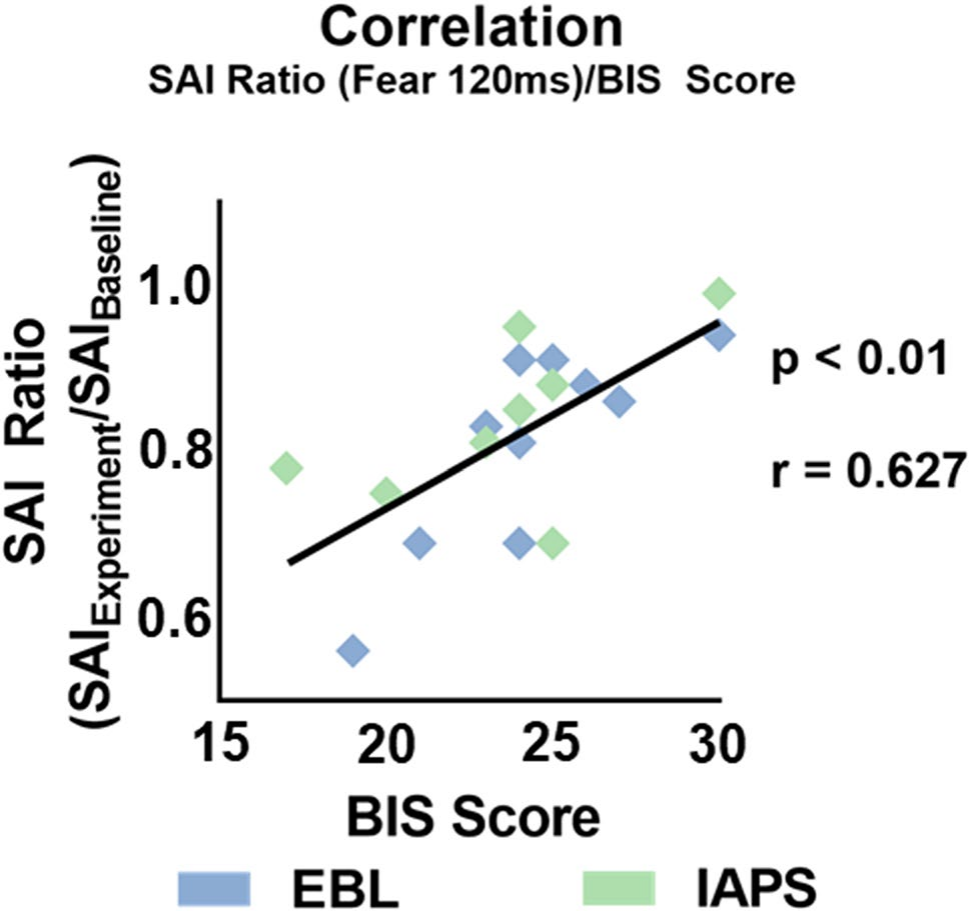
Correlation analysis on SAI Ratio and BIS. Pearson correlation analysis for SAI Ratio computed for fearful stimuli at 120 ms after stimulus onset and BIS scores of participants from both EBL and IAPS. A positive correlation is observable between the two sets of data, meaning that at a higher BIS score corresponded a decreased SAI and vice versa.

Results showed that the only significant correlation we found was a positive correlation between BIS and SAI Ratio data for fearful stimuli at 120 ms post-stimulus (p = 0.003, r = 0.627) (Fig. 4). Correction for multiple comparisons was run in accordance with the following formula from the work by Curtin and Schulz^66^:

$${\alpha }^{{\prime}}=1-{\left(1-\alpha \right)}^{\frac{1}{\mathrm{k}}},$$

where α′ will be the new alpha-value corrected for multiple comparisons, α is the value for significance previously set in our study and *k* is the number of comparisons on the same dependent variable (which in our case is equal to 10). Therefore, the new alpha-value is set at 0.005. Acknowledged that the p-value computed for the correlation between the BIS value and the SAI Ratio data for fearful stimuli at 120 ms post-stimulus is still significant after multiple comparisons correction (p = 0.003), we can say that the positive correlations we found indicates that participants with augmented SAI at 120 ms from the picture onset had a lower BIS score. All other correlations for the BIS score, as well for the BAS score, were not significant (all p > 0.05).

### Control experiment

Statistical analyses on MEPs TEST and SAI data for the control experiment showed no significant differences for CONDITION (p > 0.05), meaning that all MEPs and their relative SAI during the observation of a non-emotional visual stimulus were comparable at baseline and at 120 ms or 300 ms after the stimulus onset (see Table 2).

**Table 2:** MEPs and SAI data in the control experiment. In the table, MEPs values for the test stimulus, SAI data and SAI ratio data are reported for baseline and for the two timepoints considered in the main study (i.e., 120 and 300 ms after stimulus onset). For the SAI ratio data, no baseline is retrievable because it was already used for its computation. All values are reported as mean ± standard error of the mean (SEM).

	Control experiment		
	Baseline	120 ms	300 ms
MEP test stimulus (mV)	0.98 ± 0.03	1.00 ± 0.08	0.94 ± 0.08
SAI	0.44 ± 0.04	0.46 ± 0.04	0.47 ± 0.05
SAI ratio	NA	1.07 ± 0.05	1.07 ± 0.03

## Discussion

The main aim of this study was to investigate sensorimotor integration in healthy adults while observing EBL and IAPS pictures. Furthermore, we explored whether interindividual differences in BIS/BAS personality traits predict the magnitude of sensorimotor integration mechanisms as tapped by the SAI effect.

The results showed a consistent SAI effect on single pulse TMS when ES was administered over the median nerve. Remarkably, we observed the following sets of novel findings: (i) only while processing fearful stimuli, sensorimotor inhibition (i.e., the SAI effect) increased in magnitude, at earlier latencies (120 ms after the stimulus onset) irrespective of the type of picture (EBL or IAPS); (ii) at 300 ms after the stimulus onset, sensorimotor inhibition decreased while processing all kind of emotional stimuli (fear, happiness, neutral) again irrespective of the type of picture (EBL or IAPS); (iii) lastly, there was a correlation between sensorimotor inhibition at earlier latencies (120 ms after the stimulus onset) while processing fearful stimuli and BIS score.

There are different possible explanations accounting for the enhanced SAI effects—that is, increased sensorimotor inhibition—observed at 120 ms after the stimulus onset only for fearful stimuli, and these accounts deal with modulation of sensory afference and activity in the attentional circuits. Indeed, the circuitry underlying SAI is complex. A recent neuronal model of SAI proposes that sensory afference drives corticospinal activity through a perisomatic inhibitory projection as well as the modulation of distal dendritic excitatory input from the I-wave generators^38^. The perisomatic inhibitory input is proposed to originate from ɣ-amino butyric acid (GABA) basket cells located in layer IV of the motor cortex. These basket cells are hypothesized to receive excitatory input from somatosensory pyramidal neurons as well as from the thalamus and are sensitive to cholinergic inputs coming from the basal forebrain (BF)^67,68^. The BF cholinergic system has extensive diffuse projections to neocortex as well as projections to and from basolateral amygdala and olfactory bulb and is strongly implicated in attention^11,39^.

Studies suggest that increased sensorimotor inhibition may derive from an augmentation of the sensory afferent volleys to the cortex. Indeed, it has been consistently shown that the greater the afferent volley evoked by peripheral stimulation, the stronger is the magnitude of SAI (for a review see Ref.^38^). However, modulation of sensorimotor inhibition may also stem from the engagement of attentional circuits. Indeed, paying attention to the body part tested in the SAI protocol (hand) during an attentional task (internal focus) leads to a greater SAI with respect to what observed when the focus is external^69^. Such an effect was explained by observing that internal focus might result in an augmentation of somatosensory volleys from the specific body part, which is in keeping with the notion that observing and paying attention to a specific body part enhance the somatosensory representation of that body part^70^. In a similar vein, observing a touch onto another body—which is known to engage the activation of the somatosensory cortex through a somatosensory resonance mechanism^71,72^, also increase the SAI effect^73^. On the other hand, SAI effects may also be modulated by more general attentional mechanisms as well. In a recent study, increase of SAI was observed during a computerized non-verbal recognition memory task that requires recognizing previously encoded faces, in the retrieval phase^40^. The authors proposed that the retrieval phase of the visual memory task could evoke a considerable activation of widespread cholinergic projections from the BF to the cortex^40^.

In a more speculative way, we can also ask ourselves what is the structure that may theoretically drive these mechanisms (modulation of sensory afference and/or recruitment of attentional circuits) during processing of emotional fearful aversive stimuli? Although in our study we cannot directly address this issue, we know that a pivotal structure in decoding emotional information, specifically for threat-related stimuli, with widespread cortical and subcortical interactions is the amygdala (AMG). AMG has been shown to receive complex sensory information from the periphery and process them in its lateral and basolateral nuclei, which in turn project to its central nucleus^74^. Then, the central nucleus of the AMG sends projections to a widespread network involving a multitude of structures which are involved in both motor and non-motor responses at short and long latencies^3,4,75,76^. More specifically, in response to aversive stimuli, the central nucleus of the AMG has been shown to project to several subcortical structures involved in attentional processes such as the BF and the locus coeruleus. Through its projections toward cholinergic nuclei located in the BF, AMG is able to influence an extended cortical and subcortical network which in turn increases the attentional control over a stimulus^3,39^. Furthermore, this widespread cholinergic activity might be able not only to increase attention, but also to lower the threshold of the sensory system in order to focus attentional resources over threat-related stimuli and consequently potentiate sensory afferences processing^39^. Projections from the central nucleus of the AMG have also an influence over the noradrenergic cells of the locus coeruleus, structure responsible for stress and conditioned fear response as well as for autonomic activity^39,77,78^.

Taken all together, the engagement of cholinergic circuits secondary to emotional stimulation coupled together with the activation of the locus coeruleus and the concomitant activation of the sensory thalamus may lead to an increased state of vigilance which directs attention toward strongly arousing stimuli with aversive properties^3,11,39,77^.

Related to the temporal dimension of the activation of the AMG, it has been shown that the AMG promptly reacts to fearful signals, for example showing a short-latency activation at already ~ 70 ms from presentation of a fearful face—which might be linked to an evolutionary mechanism for processing threat-related stimuli, in order to rapidly react to a potential menace^79^. Such a mechanism was referred as ‘preparedness’, which is defined as an instinctive response to known aversive stimuli that does not show an elaborated cognitive processing of the afferent information^80^. So far this phenomenon has been described in processing emotional facial expressions^79^, but since subcortical areas activated during facial expression processing are partially overlapping with those engaged during processing of EBL and IAPS (one over all is AMG), it is plausible to expect similar pattern of activation for both categories of emotional stimuli^6,81,82^.

Based on the aforesaid premises and on our results, it appears reasonable to speculate that the rapid amygdala activation, as described for fearful facial expressions, might be a key component for the sensorimotor modulation we found for fearful pictures, acting via the same principle of ‘preparedness’ observed for threat-related faces, even though specific imaging studies are needed in order to confirm these supposed mechanisms.

Moreover, the fact that we observed such a fast increase of SAI for all fearful pictures, being them EBL or IAPS, might be related to the fact that an aversive emotional content is able to rapidly activate a vast and intertwined network capable of focusing attentional resources over a potential threat via the induction of sensory vigilance and hence inducing a transient and short-latency freezing-like phenomenon of the motor cortex^12,79^.

The second result of our study was that at longer latency (i.e., 300 ms after the stimulus onset) sensorimotor inhibition decreased while processing all kind of emotional stimuli (fear, happiness, neutral) conveyed by all the pictures (EBL and IAPS). First, it is worthy to note that in our control experiment, planned to test SAI at 120 and 300 ms after the onset of a picture representing a black cross on a white screen, we did not find a modulation of SAI. This result mostly excludes that solely being presented with a visual stimulus shown on a screen may modulate sensorimotor inhibition (for a detailed analysis on the topic see “Limitations”).

As an example of sensorimotor modulation derived from higher order cognitive processes, a decreased sensorimotor inhibition has been observed during a verbal working memory task, with increasing memory set size^42^. Indeed, SAI was significantly reduced during memory maintenance of six-digit compared to two-digit set^42^. This result was interpreted as the necessity to increase the suppression of the task-irrelevant somatosensory afferent projections to motor cortex during the maintenance period of the verbal working memory task. Following this finding, here we showed no modulation during visual processing of a blank screen, but when the visual information increased in complexity, as for IAPS and EBL pictures, SAI decreased at 300 ms. We might propose that even during passive visual processing of images of different complexity, decreased sensorimotor inhibition may represents the suppression of the task-irrelevant somatosensory afferent projections to motor cortex, similarly to what happens during a higher order cognitive processing such as a visual working memory task of increasing complexity. Indeed, 300 ms latency is consistent with cognitive processing of the visual stimuli. Event-related potentials studies showed during visual processing of a variety of stimuli a consistent component related to cognitive control, that is the P300, a positive wave that occurs at around 300–450 ms after stimulus presentation and which is maximal over the central-parietal region^83,84^. Major determinants of P300 amplitude during visual processing are reward value, affective significance and the influence of these factors on attention resource allocation^9,85,86^.

The third result of our study was that sensorimotor modulation during processing of fearful stimuli at 120 ms correlated with the BIS score: the more the increase in sensorimotor inhibition at 120 ms while processing fearful stimuli, the lower the BIS score. A low score in the BIS questionnaire indicates an individual minor propensity in withdrawal behaviour when facing a potential threat. If modulation of sensorimotor inhibition is driven by the activation of the AMG orchestrating a cascade of events including increased attention with higher sensory vigilance, we can hypothesize that these processes are linked to behaviour, including propensity in withdrawal behaviour. Greater attention and sensory vigilance may guide a higher propensity in facing a potential threat, rather than withdraw from it. This speculation is in line with recent behavioural findings of our group: by using the same sets of EBL and IAPS images adopted here and analysing response time in a recognition task, we found that response time for fear pictures were lower than for happy and neutral, particularly for EBL pictures^24^. Possible explanations for this finding may lie on shorter time for processing fear respect to happy pictures, but also on fear stimuli facilitating faster motor commands with respect to happy and neutral.

In any case it is important to note that, although the results of the correlation analysis resulted significant for multiple comparisons correction, deeper investigations are needed in order to confirm our findings with a bigger sample size and separately for EBL and IAPS.

### Limitations

Some limitations of this study deserve to be discussed. First, we a priori decided to explore sensorimotor inhibition only at 120 ms and 300 ms from picture onset, in order to test for a rapid-automatic sensorimotor modulation and for cognitive processing of EBL and IAPS. However, in future studies it will be of interest to study the temporal dynamics of sensorimotor inhibition more in the details analysing, for instance, whether an automatic process starts early for EBL or IAPS pictures and how long it lasts. Second, future studies may explore whether sensory gating or sensory augmentation mechanisms are involved, by the analysis of somatosensory evoked potentials. Moreover, a more structured online control task in order to verify that the participants were paying attention to the visual stimuli sliding on the screen (e.g., question about the emotional content of the observed stimulus between trials) might be of use in order to eliminate a possible bias regarding attentional orientation. It would also be of interest a control on visual orientation, via an eye-tracking system, able to assess whether sensorimotor inhibition in EBL is linked to the observation of a specific body part (e.g., hands) or to the body posture as a whole.

We also have to consider as a limitation the limited sample size used in the control experiment. Whether sensorimotor inhibition is not modulated by simple, non-emotional stimuli but only by complex images more attentional engaging, is worthy to be confirmed in a larger sample size.

Finally, the role of cholinergic circuits may be addressed by exploring this process in pathological conditions with cortical cholinergic denervation (i.e., Alzheimer’s disease) or under pharmacological modulation of cholinergic activity.

## Conclusions

Here we showed that sensorimotor inhibition, depending on the magnitude of afferent sensory volley and on the activity of cholinergic circuits, is dynamically modulated during visual processing of emotional stimuli. At short latency from picture onset, reflecting automatic processing of emotional information, only negative, fearful stimuli induced a modulation of sensorimotor inhibition. At 300 ms from picture onset, reflecting cognitive appraisal of visual information, SAI appeared to decrease in relation to the complexity of the image, rather than its emotional content. Sensorimotor inhibition has been used so far as a tool to explore cognitive-motor interaction, but the results of this study suggest that it may also be used to explore emotional-motor interaction, in physiological and pathological conditions.

## Supplementary Information

Supplementary Information.

## Data Availability

The data that support the findings of this study are available from the corresponding author upon reasonable request.
