# Supplementary material for: Sensorimotor inhibition during emotional processing
Source: Sci Rep. 2022 Apr 29;12:6998. doi: 10.1038/s41598-022-10981-8 (PMC9054825; doi:10.1038/s41598-022-10981-8)
Supplement: Supplementary file 1 — Supplementary Information. [file 41598_2022_10981_MOESM1_ESM.docx]

**Supplementary Materials**

**Sensorimotor inhibition during emotional processing**

*Alessandro Botta, Giovanna Lagravinese, Marco Bove, Elisa Pelosin, Gaia Bonassi, Alessio Avenanti and Laura Avanzino*

**Supplemental methods:**

**List of IAPS images used in the main Experiment**

Negative images: 1120, 1525, 1932, 2120, 5971, 5973, 6230, 6250, 6510, 6550, 6570.1, 8480, 9230, 9908, 9930. Positive images: 1441, 1463, 1710, 1999, 2035, 2045, 2057, 2224, 2314, 2340, 2347, 2530, 2550, 4623, 7325. Neutral images: 5390, 7002, 7003, 7004, 7006, 7009, 7021, 7026, 7039, 7041, 7052, 7492, 7530, 7650, 8325. All the negative stimuli were taken from the study from Barke et al. [S1].

**Characteristics of visual stimuli**

All the visual stimuli used in the main experiment were taken from a previous study from our group [S2]. In the table below are shown the ratings for valence and arousal and the percentage of accuracy in the emotional content recognition of the EBL (named ‘Postures’ in the table) and IAPS stimuli. All values are reported as Mean ± S.D. Valence and arousal were evaluated on a 1-to-9 Likert scale.

As observable in the table, all visual stimuli were matched for valence (i.e., negative stimuli = low valence, positive stimuli = high valence, neutral stimuli = valence equal to 5) and arousal (high arousing stimuli = arousal higher than 5, low arousing stimuli = arousal lower than 5) [S3].

Regarding the EBL stimuli, all the validation analyses are retrievable in the main study and in the supplementary materials provided by Borgomaneri and colleagues in the paper named “Motor mapping of implied actions during perception of emotional body language” [S4].

**Linear regression with GROUP (EBL and IAPS) as predictor of BIS/BAS and SAI Ratio data**

|  | **p** | **Standardized β** | **R^2^** | **95% C.I.** | |
| --- | --- | --- | --- | --- | --- |
| **BIS** | 0.628 | -0.123 | 0.015 | -4.229 | 2.629 |
| **BAS** | 0.681 | -0.104 | -0.051 | -5.614 | 3.764 |
| **SAI Ratio**  **Fear 120ms** | 0.592 | 0.135 | 0.018 | -0.85 | 0.144 |
| **SAI Ratio**  **Fear 300ms** | 0.549 | 0.151 | 0.023 | -0.180 | 0.326 |
| **SAI Ratio**  **Happiness 120ms** | 0.627 | -0.123 | 0.015 | -0.241 | 0.149 |
| **SAI Ratio**  **Happiness 300ms** | 0.761 | -0.077 | 0.006 | -0.264 | 0.197 |
| **SAI Ratio**  **Neutral 120ms** | 0.763 | 0.076 | 0.006 | -0.283 | 0.379 |
| **SAI Ratio**  **Neutral 300ms** | 0.787 | -0.274 | 0.005 | -0.339 | 0.262 |

**Supplemental References:**

[S1] Barke, A., Stahl, J. & Kröner-Herwig, B. Identifying a subset of fear-evoking pictures from the IAPS on the basis of dimensional and categorical ratings for a German sample. *J. Behav. Ther. Exp. Psychiatry* **43**, 565–572 (2012)

[S2] Botta, A., Lagravinese, G., Bove, M., Avenanti, A. & Avanzino, L. Modulation of Response Times During Processing of Emotional Body Language. *Front. Psychol.* **12**, 1–11 (2021)

[S3] Bradley, M. M., Codispoti, M., Cuthbert, B. N. & Lang, P. J. Emotion and Motivation I: Defensive and Appetitive Reactions in Picture Processing. *Emotion* **1**, 276–298 (2001)

[S4] Borgomaneri, S., Gazzola, V. & Avenanti, A. Motor mapping of implied actions during perception of emotional body language. *Brain Stimul.* **5**, 70–76 (2012)
